# Disordered Eating Behaviors and Insulin Restriction in Saudi Adolescents and Young Adults with Type 1 Diabetes

**DOI:** 10.3390/medicina59020345

**Published:** 2023-02-11

**Authors:** Saeed Yafei, Abdulrahman Hummadi, Mohammed Badedi, Hussain Darraj, Abdullah Khawaji, Turki Alzughbi, Raed Abutaleb, Ali Jaber Alhagawy, Awaji Alnami, Bayan Kudam, Fatma Bahsan, Maryam Kariri, Morghama Adawi, Mohammed Daghriri, Rania Hassan, Mohammed Soeid, Nouf Alzughbi

**Affiliations:** 1 Jazan Endocrinology & Diabetes Center, Ministry of Health, Jazan 45142, Saudi Arabia; 2 Faculty of Medicine and Health Sciences, Taiz University, Taiz P.O. Box 6803, Yemen; 3 Administration of Research & Studies, Jazan Health Affairs, Jazan 82611, Saudi Arabia; 4 Family Medicine Department, Ministry of Health, Jazan 45142, Saudi Arabia

**Keywords:** disordered eating behavior, DEBs, type 1 diabetes, DEPS-R, insulin restriction

## Abstract

*Background and Objectives:* The prevalence of disordered eating behaviors (DEBs) in type 1 diabetes (T1D) has been studied globally; however, data from Saudi Arabia and Arab-speaking countries are scarce. This study aimed to measure the prevalence of DEBs and associated clinical characteristics in adolescents and young adults with T1D, and the impact of DEBs on glycemic parameters. *Materials and Methods:* A total of 265 adolescents and young adults with T1D (aged 12–25 years) were recruited randomly from the registry at the Jazan Endocrinology and Diabetes Center, Saudi Arabia. Participants completed the Diabetes Eating Problem Survey–Revised (DEPS-R) questionnaire. Other measures included hemoglobin A1c (HbA1c) in addition to sociodemographic, anthropometric, and clinical data. *Results:* The prevalence of DEBs among T1D was 27.2%. Females (32.5%) had a higher proportion of DEBs than males (18.6%) (*p* = 0.01). About 27% of the participants reported insulin restriction. A history of hospital admission in the previous 6 months due to DKA and frequent hypoglycemia was more frequently reported in T1D participants with DEBs compared to those without (*p* = 0.03). Participants with DEBs had higher HbA1c and higher BMI compared to those without DEBs. *Conclusions:* Adolescents and young adults with T1D with disordered eating and insulin restriction have higher glycated hemoglobin, and are at higher risk of DKA. Routine screening for DEBs should be an essential element in diabetes care, especially among highly vulnerable groups.

## 1. Introduction

Type 1 diabetes is a form of diabetes occurring usually during childhood and adolescence due to the autoimmune destruction of beta cells of the pancreas with absolute insulin deficiency. Recent reports from 2021 estimated that about 8.4 million individuals worldwide are living with type 1 diabetes (T1D) [1]. Management of T1D is a complex psychological process that requires lifelong insulin therapy and constant strict self-management, balancing insulin doses, diet, and exercise with frequent blood glucose monitoring to maintain good glycemic control [2].

Disordered eating behaviors (DEBs) are subclinical or milder forms of eating disorders (EDs), such as unhealthy dieting, binge eating, and purging [3]. People with T1D are at high risk for DEBs, especially adolescents and young adults [4], as diabetes management essentially depends on an intense awareness of food intake, a focus on exercise, avoidance and prompt management of hypoglycemia, and sometimes the intentional insulin restriction or omission to avoid weight gain [5]. The combination of T1D and DEBs is of great concern, as DEBs are associated with poor glycemic control, high risk of hypoglycemia, frequent episodes of diabetic ketoacidosis (DKA), and accelerated serious complications [4,6].

Pinhas-Hamiel et al. [3] suggested a three-level model to describe the development of disturbed eating in adolescents and young adults with T1D. First, the premorbid state includes a tendency to become overweight and involves consideration of personality or familial characteristics. The second state includes factors arising at diagnosis of diabetes, such as the age at onset of diabetes and satisfaction from weight loss. The third state includes factors associated with the chronic management of diabetes, such as recurrent hypoglycemic episodes, strict insulin treatment, and carbohydrate counting.

A clinical interview is the gold standard for an ED diagnosis, but in most circumstances, it might be difficult and time-consuming to conduct a clinical interview for the identification and diagnosis of eating disorders in the general population or in patients with T1D. Hence, screening tools for DEBs were developed where individuals who scored above a specific cut-off on an eating disorders screening instrument are considered to be at high risk for an ED [7]. The Diabetes Eating Problem Survey–Revised (DEPS-R), developed by Markowitz et al., [8] is a diabetes-specific screening tool for DEBs composed of 16 items. It is considered the best-validated screening tool for DEBs in children, adolescents, and adults [9]. Responses are scored on a six-point Likert scale where higher scores indicate greater pathology. A recommended cut-off score of 20 or more has been proposed as a threshold indicating the need for further clinical assessment of eating disorders [8].

Saudi Arabia is one of the top 10 countries with the highest prevalence and incidence of type 1 diabetes in children and adolescents below the age of 20 [10]. Compared to the extensive work conducted on type 2 diabetes mellitus (T2DM), there is a paucity of research investigating the prevalence of T1D, and the factors predicting glycemic control among Saudi children and adults [11,12,13]. The prevalence of EDs was assumed to be low in Arab-speaking countries; however, in recent years the risk of developing an ED has been increasing, especially in females [14], as a result of sociocultural changes, globalization and urbanization, and the impact of social media [15]. The prevalence of disordered eating among T1D patients was not studied before in Saudi Arabia. Therefore, this study aimed to measure the prevalence and clinical characteristics of DEBs in Saudi adolescents and young adults with T1D, and the impact of DEBs on glycemic parameters.

## 2. Materials and Methods

### 2.1. Participants and Measures

This cross-sectional study included 265 Saudi adolescents and young adults with T1D (aged 12–25 years) on multiple insulin treatments for at least one year. Using non-probability random sampling, participants were recruited from the registry of the Jazan Endocrinology and Diabetes Center (JEDC), Saudi Arabia, between April and July 2022. Patients with type 2 diabetes, pregnant women, and patients on treatment for psychiatric illness were excluded from this study. The Jazan Health Ethics Committee (reference number: H-10-Z-073) granted ethical approval for conducting the present study, and this research complied with the Declaration of Helsinki. 

Data collection included gathering information about the patient’s age, sex, education level, age of diagnosis, and duration of diabetes. Clinical data included type of insulin therapy, usage of continuous glucose monitoring, total insulin dose, frequency of blood glucose monitoring, frequency of severe hypoglycemia, and recent admissions due to hypoglycemia or DKA in the previous six-month period. Anthropometric and biochemical data included weight and height, body mass index (BMI) measurements, fasting blood glucose, and hemoglobin A1c (HbA1c).

### 2.2. Disordered Eating Behavior (DEB)

A DEB was assessed using a valid Arabic version of the questionnaire, The Diabetes Eating Problem Survey–Revised (DEPS-R), developed by Markowitz et al. [8] and translated into Arabic by Hummadi et al. [16]. This questionnaire contains 16 items, with scoring on a 6-point Likert scale, where “0” represents “never,” and “5” represents “always.” The total DEPS-R score can range from 0 to 80, with a cutoff point at ≥20, and with a higher score indicating a higher risk for an ED [8].

In line with previous studies [8,17], we defined insulin restriction and insulin omission based on these two DEPS-R items: “When I overeat, I do not take enough insulin to cover the food” and “After I overeat, I skip my next insulin dose.” Answers indicating reducing or skipping “at least sometimes” over the previous few months were classified as insulin restriction or omission.

### 2.3. Statistical Analysis

Data entry and analysis were performed using the Statistical Package for the Social Sciences software. The normality of the distribution of variables was determined visually and using the Shapiro–Wilk test. Categorical variables are presented as count and percentage values, while continuous variables are presented as the mean ± standard deviation (SD) for normally distributed variables and as the median (interquartile range = IQR) for non-normally distributed variables. 

The chi-square test was used to detect any significant associations between categorical variables. Pearson’s coefficient was used to assess the correlation between the DEPS-R score and other variables for normally distributed variables, while Spearman’s Rho was used for non-normally distributed variables. The independent samples t-test and Mann–Whitney U-test were used to compare differences in the mean values of normal and non-normal variables, respectively, between groups (adjusted for sex, age, and BMI). For assessment of effect size, Cohen’s d for the t-test was used (>0.2 small, >0.5 medium, >0.8 large) [18]. Significance was set at *p* < 0.05.

## 3. Results

### 3.1. Clinical and Anthropometric Characteristics 

The current study recruited 265 Saudi participants with T1D. The categorization and distribution of the groups are presented in Table 1. Adolescents represented 53.9% of the total participants, while young adults represented 46.1% (Table 1). There were more females (61.5%) than males (38.5%) in the studied population. The mean age (SD) of all participants was 17.7 (3.5) years, which ranged from 12 to 25 years. The median duration of diabetes was 6 years (range: 1–21 years), and the median age at diagnosis was 11 years (1st–3rd quartiles: 8–13). Depending on the adjusted BMI, 38 of the participants were classified as underweight (14.3%), 183 were of normal weight (69.1%), 27 were overweight (10.2%), and 17 were obese (6.4%). Other sociodemographic and clinical features of the participants are provided in Table 1 and Table 2.

Based on the sex group comparison, females had higher BMI than males (*p* < 0.001, d = 0.42), and reported higher usage of fingerstick testing for measuring blood sugar (*p* = 0.007) (Table 1). About 12.9% of females reported DKA admissions in the previous six months, compared to 5.9% of males (*p* = 0.01). Severe hypoglycemia events were also more prevalent in females (19%) compared to males (6.9%) (*p* = 0.006). No significant differences between the genders were found for age, diabetes duration, age at onset of diabetes, HbA1c levels, education level, use of carb counting, and insulin omission or restriction (*p* > 0.05) (Table 1).

Comparing adolescents and young adults, about 37.1% of adolescents had HbA1c < 7.5%, while 37.7% of young adults had HbA1c < 7.0%. About one-third of the total sample (31.7%) had poor glycemic control (HbA1c > 9.0%). Adolescents reported higher usage of CGM (84.6%; *p* < 0.001) and lower usage of fingerstick for SMBG (63.6%; *p* = 0.027) compared to the young adults (Table 1). No significant differences between the age groups were found in terms of BMI, HbA1c values, dependency on carb counting, or history of recent admission for DKA or hypoglycemia in the previous 6 months (all *p* > 0.05) (Table 1). 

### 3.2. Disordered Eating Behaviors

The overall DEPS-R mean score was 15.4 ± 10.7. Of all participants, 27.2% scored ≥ 20 on the DEPS-R, indicating a high risk for the development of DEBs. Females (32.5%) had a significantly higher DEBS-R score of 16.8 ± 10.9 than that for the males (18.6%), which was 13.3 ± 10 (*p* = 0.01). In a comparison between adolescents and young adults, the proportion of participants with a DEPS-R score ≥ 20 was similar in both groups, and there was no difference in the mean score (Table 1).

Table 2 compares the different features of those with and without DEBs. Participants with DEBs had higher HbA1c (*p* < 0.001, d = 0.87) and higher BMI values (*p* < 0.001, d = 0.70). A history of hospital admission due to DKA in the previous 6 months was more frequently reported in patients with DEBs compared to those without DEBs (16.7% vs 7.8%, respectively; *p* = 0.033). Participants with DEBs reported a higher frequency of severe hypoglycemia (27.8%; *p* < 0.001) and more frequent admissions due to severe hypoglycemia (9.7%; *p* = 0.048). Further analysis of those with and without DEBs revealed no significant differences in age, diabetes duration, age at onset of diabetes, family history of diabetes, education level or family education, and the use of carb counting or CGM (all *p* > 0.05) (Table 2). 

Based on the bivariate correlation of the DEPS-R score with other variables, we found that the total DEPS-R score was correlated with HbA1c (females: r = 0.365; males: r = 0.492; *p* < 0.001 in both) and BMI (females: r = 0.424, *p* < 0.001; males: r = 0.225, *p* = 0.023). The DEPS-R correlated weakly with age at diagnosis in males only (r = 0.201, *p* = 0.043). No correlation was found with age or duration of diabetes for either gender.

### 3.3. Insulin Restriction

A total of 27.5% of the participants restricted their insulin doses, and 12.1% skipped their insulin doses at least occasionally after overeating (Table 2). Insulin restrictors had significantly higher HbA1c (9.2 ± 2.2; *p* < 0.001), later onset of diabetes (11.6 ± 4.5; *p* = 0.041), and significantly higher DEPS-R scores (24.8 ± 10.4; *p* < 0.001) (Table 3). About 59.7% of insulin restrictors scored ≥ 20 on the DEPS-R compared with 15.1% of non-restrictors (*p* < 0.001). There was no statistically significant difference in insulin restriction or omission across genders or age groups. Regarding gender, 29.4% of females and 24.5% of males reported restricting insulin when overeating, and 13.5% of females skipped their insulin doses at least occasionally, compared to 9.8% of males (*p* > 0.05 in both). Across the age groups, 26.5% of adolescents and 29.3% of young adults reported insulin restriction, and 11.4% of adolescents reported insulin omission, compared to 13.1% of young adults (*p* > 0.05 in both).

## 4. Discussion

The present study estimated the prevalence of DEBs and insulin restriction in a sample of Saudi adolescents and young adults with T1D using the diabetes-specific DEPS-R scale. This study included adolescents and young adults with T1D, as these ages are considered important risk periods for eating disorders and DEBs [7]. This is a critical period of human development, during which several physical, cognitive, and behavioral changes occur, including an increased incidence of risky behaviors such as unhealthy dietary behaviors [4]. Furthermore, eating behavior often becomes less healthy during the transition from adolescence to young adulthood; thus, DEBs are not just an adolescent problem but continue to be prevalent among young adults [19].

The prevalence of DEBs in this study was 27.2%, which is comparable to the prevalence previously reported in T1D from Norway (18.3%) [20], Italy (34.4%) [21], and the United States (21.2%) [22]. To the best of our knowledge, this is the first report using the DEPS-R scale on the prevalence of DEBs in the Saudi population with T1D. In this regard, and upon reviewing the existing literature from the Arab world, only minimal data are available. A recent study [23] on 138 young Egyptian patients with T1D found that 17.4% of the total sample scored positive for DEBs on the eating attitude test, while 32.6% scored positive for DEBs on the Eating Disorder Examination Questionnaire (EDE-Q6) [9]. 

There is general agreement that females are at greater risk than males for DEBs, regardless of the presence of underlying disease [24]. The development of T1D during preadolescence seems to place already susceptible girls at even greater risk for the subsequent development of eating disorders [25]. Furthermore, both social media and peers influence adolescents’ body image, increasing the risk for DEBs, especially in females [26]. In line with this evidence, the prevalence of DEBs in females was 32.5%, compared to 18.6% in males. These findings do not differ from previous reports from different countries using the DEPS-R scale in T1D patients. For example, Nip et al. [22] found that 30.8% of females aged 13–25 years with T1D have DEBs. Similarly, Troncone et al. [27] and Wisting et al. [20] found that the proportion of adolescent females with T1D who had DEBs was 35% and 27.7%, respectively.

Our study found no significant difference in DEB scores in adolescents (27.7%) and young adults (26.3%) (*p* > 0.05). While such results are interesting, this needs to be explored further in future research. Most of the available literature on DEBs studied adolescents or adults separately, and only a few studies compared both groups together [22,28]. Rancourt et al. [28] reported that 29.64% of T1D adolescents and 34.50% of T1D young adults have DEBs (*p* = 0.017). In a 10-year longitudinal study tracking eating behavior from adolescence to young adulthood, Neumark et al. [19] found that the prevalence of disordered eating was high and remained constant, or increased, from adolescence to young adulthood. Although ours is not a longitudinal study, we can conclude that adolescents and young adults with T1D are at high risk of EDs.

In line with previous studies [17,21,29], participants with DEBs in this study had higher HbA1c, higher BMI values, more frequent histories of hospital admission due to DKA, and more frequent episodes of severe hypoglycemia. Furthermore, based on the results of the correlation analysis, the mean DEPS-R score was correlated with HbA1c and BMI. Several review articles on DEBs in adolescents with T1D describe the association between DEBs, glycemic control, and BMI [3,4,9,24]. Generally, the relationship between DEBs, BMI, and HbA1c is complicated. Higher body weight is always a major concern in individuals with T1D since there is a strong relationship between food intake, insulin therapy, and glycemic control. Higher BMI has been hypothesized to lead to increased body dissatisfaction and a greater desire to lose weight, which may result in dieting, negative affect, and disordered eating [27]. Pinhas-Hamiel [3] hypothesized that food preoccupation imposed by carbohydrate counting, weight fluctuations associated with variable use of insulin, and the subsequent body and blood glucose fluctuations associated with mismatched insulin dose, as well as excessive caloric intake secondary to hypoglycemia, can burden those with T1D and may increase their vulnerability to the development of DEBs. 

Insulin omission or restriction is another common behavior in patients with T1D and involves skipping or restricting insulin doses for different reasons, including weight loss [30,31]. The prevalence of insulin restriction in our study was 27.5%, and another 12.1% of the participants reported skipping their insulin dose entirely at least occasionally after overeating. The prevalence of insulin restriction in the existing literature differs significantly [32]. Wisting et al. [20] reported that 31.6% of adolescents with T1D restrict their insulin doses and 6.9% skip a dose at least occasionally. In a national survey of Australian adolescents with T1D [33], intentional insulin omission was reported in 19% of adolescents, regardless of gender. The SEARCH for Diabetes in Youth study [22] found that about 18% of adolescents and young adults with T1D skip their insulin doses. 

We found no statistically significant differences between males and females regarding the frequency of insulin omission or restriction. In this regard, several studies reported no gender difference in intentional insulin restriction or omission [20,21,33,34], while other studies found that insulin omission is higher in females [35,36]. Furthermore, and in line with previous evidence [17,20,33], we found that adolescents and young adults who restrict insulin have higher HbA1c levels and higher DEPS-R scores. This important finding in the current study has a direct clinical implication, as poor glycemic control is linked to an increased incidence of DKA and other metabolic or vascular complications of diabetes. 

It is important to note that the two DEPS-R questions concerning insulin restriction and omission do not specify that the reason for such behavior is for the purpose of weight loss. As this study could not identify this reason, the results should be interpreted cautiously. Generally, insulin omission is reportedly performed mainly for weight loss, but there are other possible reasons for insulin reduction or omission, including injection anxiety, fear of hypoglycemia, interference with daily living activities, or as a result of diabetes burnout due to lack of treatment adherence [30].

In summary, this study is the first Saudi study that used the diabetes specific DEPS-R score. This study has identified relatively high rates of both DEBs and insulin restriction among adolescents and young adults with T1D. The observed association between DEBs, BMI, and high HbA1c and more frequent DKA or severe hypoglycemia should raise physician awareness about routine screening for eating problems and insulin restriction especially among those with poor glycemic control or high BMI to prevent complications [20]. Insulin restriction is a common weight loss strategy in T1D patients, but other causes of insulin omission or restriction should be ruled out and approached accordingly.

Our study has some limitations. Firstly, as this is not a population-based study, the results may be different when incorporating a larger group of T1D patients of different age groups, from different regions of Saudi Arabia, or from different Arab countries. This is not a longitudinal study and does not, therefore, allow for the estimation of age at onset of eating disorders. Since a clinical interview is the gold standard for the diagnosis of eating disorders and DEBs, the use of a self-administered questionnaire, with all of its shortcomings, should be considered another limitation, especially when investigating eating disorders across genders or age groups.

## 5. Conclusions

Adolescents and young adults with type 1 diabetes, especially females and those with high body mass index, are at greater risk of disordered eating behaviors, insulin restriction, and poorer glycemic control. Routine screening for DEBs should be an essential element of diabetes care, especially in adolescent and young adults. 

## Figures and Tables

**Table 1:** Demographic and clinical characteristics grouped by gender and age.

	Sex			Age Groups		
Variables	Female *n* = 163	Male *n* = 102	*p* Effect Size	Adolescent *n* = 143	Young Adult *n* = 122	*p* Effect Size
Male sex, *n* (%)	-	-	*-*	60 (42)	42 (34.4)	0.209
Age, years, mean (SD)	17.96 (3.7)	17.2 (3.2)	0.080	15 (1.8)	20.8 (2.2)	<0.001 *
HbA1c, mean (SD)	8.5 (1.97)	8 (2.3)	0.081	8.5 (2.2)	8.1 (2)	0.134
Duration in years, mean (SD)	7.4 (4.8)	6.4 (4.7)	0.079	5.2 (3.9)	9.2 (4.9)	<0.001 *
Age at onset, years, mean (SD)	10.5 (4.5)	10.8 (4)	0.574	9.8 (3.7)	11.6 (4.8)	<0.001 *
Insulin dose, unit/Kg/day, mean (SD)	1.1 (0.4)	1.1 (0.4)	0.616	1.1 (0.4)	1.1 (0.4)	0.730
BMI, kg/m^2^, mean (SD)	22 (4.6)	20.2 (3.7)	<0.001 * d = 0.42	20.7 (4.2)	22 (4.4)	0.017 * d = 0.297
BMI class, *n* (%)						
Underweight/Normal weight	129 (79.1)	92 (90.2)	0.019 *	119 (83.2)	102 (83.6)	0.932
Overweigh/Obese	34 (20.9)	10 (9.8)		24 (16.8)	20 (16.4)	
Education level, *n* (%)						
Low education	52 (31.9)	41 (40.2)	0.169	80 (55.9)	13 (10.7)	<0.001 *
High education	111 (68.1)	61 (59.8)		63 (44.1)	109 (89.3)	
Family education level, *n* (%)						
Low education	57 (35)	41 (40.2)	0.391	46 (32.2)	52 (42.6)	0.079
High education	106 (65)	61 (59.8)		97 (67.8)	70 (57.4)	
Family history of diabetes, *n* (%)	97 (59.5)	51 (50)	0.129	67 (46.9)	81 (66.4)	<0.001 *
CGM use, *n* (%)	116 (71.2)	77 (75.5)	0.441	121 (84.6)	72 (59.0)	<0.001 *
SMBG using fingerstick, *n* (%)	123 (75.5)	61 (59.8)	0.007 *	91 (63.6)	93 (76.2)	0.027 *
Carb counting, *n* (%)	48 (29.4)	23 (22.5)	0.217	33 (23.1)	38 (31.1)	0.139
DKA history in 6 months, *n* (%)	21 (12.9)	6 (5.9)	0.01 *	17 (11.9)	10 (8.2)	0.322
Recent severe hypoglycemia, *n* (%)	31 (19)	7 (6.9)	0.006 *	22 (15.4)	16 (13.1)	0.599
Admission due to hypoglycemia in the last 6 months, *n* (%)	9 (5.5)	5 (4.9)	0.82	8 (5.6)	6 (4.9)	0.806
DEPS-R score, mean (SD)	16.8 (10.9)	13.3 (10)	0.010 * d= 0.33	15.3 (11.5)	15.6 (9.7)	0.774
DEPS-R ≥ 20	53 (32.5)	19 (18.6)	0.013 *	41 (28.7)	31 (25.4)	0.552
Insulin restriction, *n* (%)	48 (29.4)	25 (24.5)	0.381	43 (30.1)	30 (24.6)	0.320
Insulin omission, *n* (%)	23 (14.1)	9 (8.8)	0.199	17 (11.9)	15 (12.3)	0.919

Data are presented as number (n) and mean and standard deviation (SD); Body mass index (BMI); Continuous glucose monitoring (CGM); Self-monitoring of blood glucose (SMBG); Diabetic ketoacidosis (DKA); Diabetes eating problem survey–Revised (DEPS-R); * *p* is significant at the 0.05 level.

**Table 2:** Comparison of participants with and without DEB.

Variables	Total (*n* = 265)	DEB (*n* = 72)	No DEB (*n* = 193)	*p,* Effect Size
Male sex, *n* (%)	102 (38.5)	19 (26.4)	83 (43)	0.013 *
Age in years, mean (SD)	17.7 (3.5)	17.8 (3.3)	17.63 (3.6)	0.787
HbA1c, mean (SD), %	8.3 (2.1)	9.6 (2.2)	7.9 (1.9)	<0.001 *, d = 0.87
Duration, mean (SD), years	7 (4.8)	6.7 (5)	7.1 (4.7)	0.567
Age at onset in years, mean (SD)	10.6 (4.3)	11 (4.4)	10.5 (4.2)	0.402
Insulin dose, unit/Kg/day, mean (SD)	1.1 (0.4)	1 (0.4)	1.1 (0.41)	0.120
BMI, kg/m^2^, mean (SD)	21.3 (4.3)	23.4 (5.1)	20. (3.7)	<0.001 *, d = 0.69
BMI class, *n* (%)				
Underweight/Normal weight	221 (83.4)	46 (63.9)	175 (90.7)	<0.001 *
Overweigh/Obese	44 (16.6)	26 (36.1)	18 (9.3)	
Poor glycemic control, *n* (%)	166 (62.6)	63 (87.5)	103 (53.4)	<0.001 *
Education level, *n* (%)				
Low education	93 (35.1)	27 (37.5)	66 (34.2)	0.616
High education	172 (64.9)	45 (62.5)	127 (65.8)	
Family education level, *n* (%)				
Low education	98 (37)	29 (40.3)	69 (35.8)	0.497
High education	167 (63)	43 (59.7)	124 (64.2)	
Family history of diabetes, *n* (%)	148 (55.8)	37 (51.4)	111 (57.5)	0.372
CGM use, n (%)	193 (72.8)	51 (70.8)	142 (73.6)	0.655
SMBG by fingerstick, *n* (%)	184 (69.4)	48 (66.7)	136 (70.5)	0.550
Carb counting, *n* (%)	71 (26.8)	14 (19.4)	57 (29.5)	0.099
DKA history in 6 months, *n* (%)	27 (10.2)	12 (16.7)	15 (7.8)	0.033 *
Recent severe hypoglycemia, *n* (%)	38 (14.3)	20 (27.8)	18 (9.3)	<0.001 *
Admission due to hypoglycemia in the last 6 months, *n* (%)	14 (5.3)	7 (9.7)	7 (3.6)	0.048 *
Insulin restriction, *n* (%)	73 (27.5)	43 (59.7)	30 (15.5)	<0.001 *
Insulin omission, *n* (%)	32 (12.1)	27 (37.5)	5 (2.6)	<0.001 *

Data are presented as number (n) and mean and standard deviation (SD); Body mass index (BMI); Continuous glucose monitoring (CGM); Self-monitoring of blood glucose (SMBG); Diabetic ketoacidosis (DKA); * *p* is significant at the 0.05 level.

**Table 3:** Comparison of insulin restrictors and non-restrictors.

Variables	Restrictors (*n*= 73)	Non-Restrictors (*n*= 192)	*p,* Effect Size
Age in years, mean (SD)	17.7 (3.4)	17.7 (3.4)	0.929
BMI, kg/m^2^, mean (SD)	22.1 (4.6)	21 (4.2)	0.077
HbA1c, mean (SD)	9.2 (2.2)	8 (2)	<0.001 *, d = 0.60
Duration in years, mean (SD)	6.1 (4.6)	7.4 (4.8)	0.064
Age at onset in years, mean (SD)	11.6 (4.5)	10.3 (4.2)	0.041 *, d = 0.29
DEPS-R score, mean (SD)	24.8 (10.4)	11.8 (8.4)	<0.001 *, d = 1.4

Data are presented as mean and standard deviation (SD); Body mass index (BMI); Diabetes eating problem survey–Revised (DEPS-R) * *p* is significant at the 0.05 level.

## Data Availability

The data presented in this study are available on reasonable request from the corresponding author.
